# Female Sexual Function and Its Association with the Severity of Menopause-Related Symptoms

**DOI:** 10.3390/ijerph17197235

**Published:** 2020-10-03

**Authors:** Isabel Pérez-Herrezuelo, Agustín Aibar-Almazán, Antonio Martínez-Amat, Raquel Fábrega-Cuadros, Esther Díaz-Mohedo, Rosemary Wangensteen, Fidel Hita-Contreras

**Affiliations:** 1 Department of Obstetrics and Ginecology, Hospital Universitario Virgen de las Nieves, 18014 Granada, Spain; iherrezuelo@yahoo.es; 2 Department of Health Sciences, Faculty of Health Sciences, University of Jaén, 23071 Jaén, Spain; amamat@ujaen.es (A.M.-A.); rfabrega@ujaen.es (R.F.-C.); rwangens@ujaen.es (R.W.); fhita@ujaen.es (F.H.-C.); 3 Department Physiotherapy, University of Málaga, 29071 Málaga, Spain; estherdiaz@uma.es

**Keywords:** sexual function, menopausal symptoms, depression, psychological, urogenital

## Abstract

The aim of this study was to examine female sexual functioning and its association with the impact of the symptoms of menopause among Spanish postmenopausal women. A total of 182 postmenopausal women (65.59 ± 7.93 years) participated in this cross-sectional study. The female sexual function index (FSFI) and the menopause rating scale (MRS) were used to analyze sexual function and severity of menopausal symptoms, respectively. Age, education, area of residence, occupation, and depression (Hospital Anxiety and Depression Scale) were considered as possible confounders. The results of a linear multivariate regression analysis showed that the severity of urogenital menopause-related symptoms was associated with lower values in the FSFI total score and the lubrication, satisfaction, arousal, and orgasm domains. These last three subscales were also linked to severe psychological impact, while the MRS total score was only related to the desire domain. Regarding confounders, being younger, working, and residing in a rural area were all linked to better sexual function. All effect sizes were large (adjusted R^2^ > 0.35). In conclusion, after controlling for possible confounders, postmenopausal women who experience a severe impact of menopausal symptoms endure poorer sexual function, particularly when said symptoms are urogenital or psychological in nature.

## 1. Introduction

Menopause represents a women’s transition from a reproductive to a non-reproductive status, and it is diagnosed after 12 months of amenorrhoea resulting from the permanent cessation of ovarian function [1]. Population aging is a global problem, affecting both developed and developing countries. With increased longevity, women live about one third of their life in the postmenopausal period [2]. It is estimated that, by 2030, a total of 1.2 billion women will find themselves in either the menopausal or postmenopausal status [3].

Postmenopausal symptoms commonly include hot flashes, night sweats (80% of women experience vasomotor symptoms during menopause), fatigue, pain, irritability, vaginal dryness, and mood changes [4,5]. Certain postmenopausal symptoms may persist for a long time [6]. Menopause symptoms have been associated with poorer quality of life [7], worse self-rated health, lower productivity at work, and increased use of public health care resources [8,9].

According to the World Health Organization (WHO), sexual health is defined as “a state of physical, emotional, mental, and social well-being in relation to sexuality; it is not merely the absence of disease, dysfunction, or infirmity” [10]. Female sexual function is a complex biopsychosocial phenomenon involving several biological, sociocultural, psychological, and interpersonal factors [11].

Female sexual dysfunction is common, affecting 25–43% of women, a percentage that increases markedly during the climacteric years [12]. It is one of the main health concerns regarding postmenopausal women, and the frequency of these sexual problems increases as menopause approaches, reaching a peak in the postmenopausal years [13,14]. Although 33–50% of middle-aged women display some degree of sexual dysfunction linked to aging and hormonal status, this percentage may vary according to several factors such as the population under analysis, the design of the study, or the approach it employs [15].

Almost two thirds of American women aged 65–74 and one half of those ≥75 years, report that sex is an important part of their lives. Even higher percentages declare that satisfactory sexual relations are essential to the maintenance of a relationship [16]. However, there are not many studies on female sexual functioning that include this age group [17].

The aim of the present study was to analyze female sexual function among Spanish middle-aged and older postmenopausal women, and to explore its association with the severity of the menopause-related symptoms.

## 2. Materials and Methods

### 2.1. Study Design and Participants

**Figure 1:**
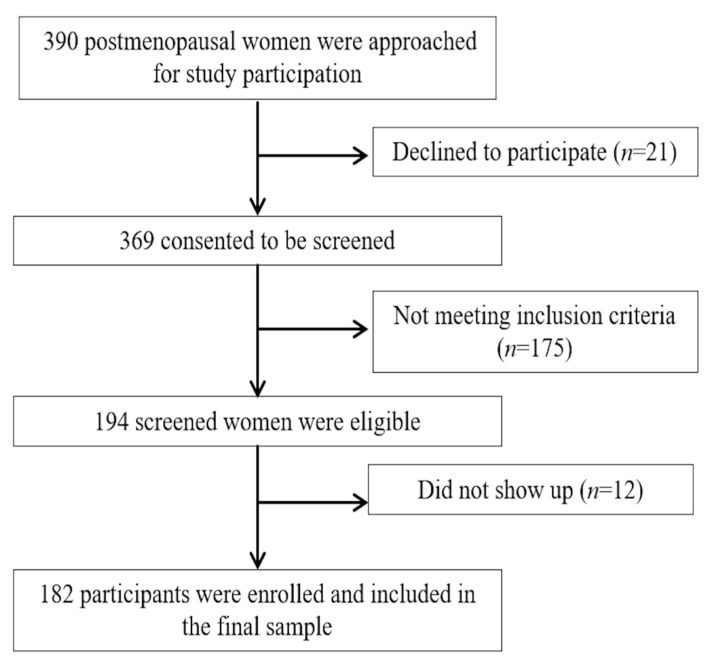
Flow diagram of study design.

An analytical cross-sectional study was conducted on 182 postmenopausal women. Participants were recruited by contacting the staff of several associations of postmenopausal women from Granada and Jaén (Spain). Figure 1 shows a flow-chart diagram of the study participants and their characteristics. This study was approved by the Research Ethics Committee of the University of Jaén, Spain (NOV.18/1.TES). All participants gave their written informed consent, and the study was conducted in accordance with the Declaration of Helsinki, good clinical practices, and all applicable laws and regulations. Inclusion criteria were at least twelve months of amenorrhoea, being sexually active in the previous four weeks (any type of sexual activity, including the full diversity of partner types, was considered), able to understand the instructions and complete the questionnaires, and willing to provide their written informed consent to participate in the study. Exclusion criteria were being under hormonal replacement therapy and suffering from any chronic and/or severe medical diseases or any neuropsychiatric disorder that might influence their responses to the questionnaire.

### 2.2. Sociodemographic and Anthropometric Data

All women were questioned by well-trained interviewers, who collected demographic and clinical data such as age, occupational status (working/not working), education (primary or less/secondary or higher), and rural (<150 inhabitants per km^2^) [18] or urban area of residence.

### 2.3. Female Sexual Function

The Female Sexual Function index (FSFI), a commonly used multidimensional questionnaire, was employed to assess sexual function [19]. It is composed of 19 items concerning the last 4 weeks, which are summarized into 6 domains or subscales: sexual (2 items); desire (4 items); arousal (4 items); lubrication (3 items); orgasm (3 items); satisfaction (3 items); and pain. Domain scores range from 0 (or 1) to 5, and the sum of the scores of all domains generates a total which indicates better sexual function the higher the score. In this study we used the validated version of the Spanish FSFI, with a score <24.95 representing a high risk of sexual dysfunction [20].

### 2.4. Severity of Menopause-Related Symptoms

The menopause Rating Scale (MRS) [21] was used to assess the severity of menopause-related symptoms and its impact on health-related quality of life. This is a self-reported questionnaire comprising 11 items grouped into 3 different subscales: somatic (4 items); psychological (4 items); and urogenital (3 items). It also provides an MRS total score. Higher scores represent increased severity of menopause-related symptoms. The cut-off points used to assess severity were ≥9 (somatic); 7 (psychological); 4 (urogenital); and 17 (total). We used the Spanish version of the MRS [22].

### 2.5. Depression

The Hospital Anxiety and Depression Scale (HADS) [23] is used to assess depressive symptoms in the general population. The HADS consists of 7 items in the depression subscale and 7 more that measure anxiety. The scoring of each item ranges from 0 (no distress) to 3 (most distress), and the total HADS depression score ranges from 0 to 21. A cut-off of ≥11 was employed to identify cases of depression. The Spanish version of the HADS was used in this study [24].

### 2.6. Sample Size Calculation

According to Concato et al. [25], in a multivariate lineal regression model at least 20 subjects per event are required for an adequate sample size. In total, 4 independent variables (MRS somatic, psychological, and urogenital domains, as well as MRS total score), together with 5 possible confounders (age, education, area of residence, occupation, and depression) were used in this study, and hence 180 participants were required. The final number of participants was 182.

### 2.7. Data Analysis

Data management and analysis were performed using the SPSS statistical package for the social sciences for Windows (SPSS Inc., Chicago, IL, USA). Categorical variables were presented as frequencies and percentages, whereas continuous variables were described using means and standard deviations. The Kolmogorov–Smirnov test was used to evaluate normality. The Student’s *t* test was performed to explore potential differences in FSFI domains and total score according to the severity of MRS domains and total scores (independent variables), as well as other confounders such as education, area of residence, occupation, and depression. Individual associations between FSFI scores and age were evaluated through a bivariate correlation analysis. In order to analyze the multivariate independent associations between variables a multivariate linear regression model was used, with FSFI scores as dependent variables. Any independent variable and confounder exhibiting significant results (*p* < 0.05) in the bivariate analysis and Student’s *t* test were included in the multivariate linear regression. In order to calculate the effect size coefficient of multiple determinations in the linear models, we employed adjustedR^2^. According to Cohen [26], adjusted R^2^ can be classified as insignificant when <0.02, small if between 0.02 and 0.15, medium if between 0.15 and 0.35, and large if >0.35. A 95% confidence level was used (*p* < 0.05).

## 3. Results

Table 1 displays the sociodemographic and clinical characteristics of our participants. Out of the 182 women (65.59 ± 7.93 years), 7.14% presented depressive mood (HADS score of 4.99 ± 3.44). Regarding FSFI, the lowest scores were recorded in the arousal and desire domains, while satisfaction was rated the highest. A total of 140 participants (70.88%) presented sexual dysfunction according to the cut-off points defined above.

When analyzing the impact of menopausal-related symptoms, 31.32% of participants reported severity in the MRS total score, while the percentages of severity for the MRS domains were 14.84%, 20.33%, and 44.51% for somatic, psychological, and urogenital symptoms, respectively. The individual associations with female sexual function (Table 2) revealed that an increased impact of psychological and urogenital symptoms was associated with worse sexual function (except for the pain domain), while the MRS total score was only related to desire. No associations were found between the somatic domain of the MRS and the FSFI scores.

Regarding depression (Table 3), women with more severe symptoms showed worse sexual function (desire, arousal, lubrication, and FSFI total scores). As for the confounder variables (data not shown), FSFI domains and total scores showed inverse correlations (all *p* < 0.001) with age, with Pearson’s correlation coefficients ranging from −0.512 (pain) to −0.689 (arousal). FSFI scores were significantly better (all *p* < 0.001) in participants who reported to be working and who had received secondary education or higher (all domains and total score), as well as in those who came from rural areas (except for the desire and lubrication domains).

Finally, a linear multivariate regression analysis was used to detect any variable independently related to decreased female sexual function (Table 4). Older age was independently related to lower values in all FSFI domains and its total score, while worse sexual function was observed in women who were not working (in the arousal, orgasm, pain, and FSFI total scores) and came from urban areas (all FSFI scores except for the desire and lubrication domains). When focusing on the MRS, the severity of menopause-related urogenital symptoms remained associated with poor sexual functioning concerning the lubrication, satisfaction, arousal, orgasm, and FSFI total scores. These last three were also linked to a severe psychological impact of the symptoms, while the MRS total score was related to the desire domain of the FSFI. Lastly, and regarding the pain domain of the FSFI, there were no independent associations with MRS scores. All effect sizes were large (adjusted R^2^ > 0.35).

## 4. Discussion

The objective of this study was to analyze whether the severity of menopausal symptoms could be independently associated with female sexual functioning among Spanish postmenopausal women. Our findings indicate that, after adjusting for potential confounding variables, a severe impact of menopausal symptoms is associated with decreased female sexual function. More precisely, in the FSFI, low arousal, orgasm, and overall scores were independently related to the severe impact of psychological and urogenital menopausal symptoms. The latter was also associated with poorer sexual function regarding lubrication and satisfaction. MRS total score was linked to lower scores in the desire domain of FSFI.

The results of our study reveal that 31.32% of participants reported experiencing a severe overall impact of menopausal symptoms (MRS total score < 17), which is in accordance with results described by Dąbrowska-Galas et al. [27] for Polish women. Meanwhile, Neutzling et al. [28] reported moderate/severe menopausal symptoms (MRS total score ≥ 9) in 57.5% and 55.2% of their participants (in age groups 50–59 and 60–69, respectively). With respect to the MRS domains, urogenital function was the more severely affected in our study, which is in agreement with results described by other authors such as Dąbrowska-Galas et al. [27], or Larroy et al. [29], who found similar results in a Spanish population. However, among Mexican women the severity of urogenital symptoms reached a much lower percentage. According to the authors of those studies, such differences may arise, in addition to cultural and social disparities, from the fact that the Mexican subjects in their study received hormone therapy and alternative therapies to a higher extent than their Spanish counterparts in order to alleviate the symptoms of menopause.

Sexual function constitutes an important part of women’s lives, and low sexual function has an adverse impact on both physical and psychological health. The FSFI is considered the gold standard in the assessment of female sexual function, as it is able to contemplate the multidimensional nature of sexual function by means of its domains or subscales. In the present study, the most highly rated aspect of female sexual function was satisfaction, while arousal and excitation obtained the lowest average scores. These findings are in agreement with the results of previous studies [30,31]. The mean ± standard deviation for the FSFI total score was 18.22 ± 10.60, which is similar to reports made by Jamali et al. [32] for postmenopausal Iranian women (60.10 ± 6.89 years), and by Gozuyesil et al. [33] (18.8 ± 8.7). However, we must bear in mind that the latter assessed sexual function only among Turkish women aged 40–60. Be that as it may, the mean FSFI total score was higher in other studies dealing with subjects of similar mean age, such as those by Carranza-Lira et al. [31] (with a score of 22) or Zhang et al. [34] (21.52 ± 2.85), after assessing Mexican and Chinese women, respectively. The differences may be due to the specific culture or the mean age of the participants. In any case, it has been shown that physical, mental, and emotional changes associated with the aging process may affect sexual function, and that their effects add up to those of menopause [35].

In our study 70.88% of participants presented sexual dysfunction, which is a lower frequency than reported in previous studies employing the FSFI and carried out among postmenopausal women of similar mean age [32,36]. Such difference may be attributable to the fact that those studies employed the traditional FSFI cut-off point described in a study that dealt with several samples of women of a wide range of ages (18–74 years) [37], while we used a cut-off point specifically indicated for an older population [20].

Sexual function is a multidimensional phenomenon, and each one of its aspects may in turn be affected by several factors. In our study, older age remained associated with all domains and the total score of the FSFI in the multivariate analysis, which is consistent with previous reports [17,38]. Blümmel et al. [39], in a multicenter study carried out across 11 Latin American countries, reported that older age (over 48 years) was associated with a higher incidence of sexual dysfunction. As a matter of fact, the mean age in that study was markedly lower than in our sample, and their scores for every domain as well as for the total score of FSFI were higher. Consistently with previous research [38,40,41], our results also revealed links between sexual function and other sociodemographic indicators. As a matter of fact, poorer female sexual function was associated with not working status and residing in an urban area, as well as with having received a primary education or less, although the latter effect did not persist in the multivariate analysis.

Menopause-related symptoms have been linked to reduced mental and physical quality-of-life scores, with a negative impact on personal and intimate relationships, occupational productivity, and activities of daily living [42,43]. Some studies have looked into the associations between the severity of menopausal symptoms and female sexual function [39,44,45], although as a general rule they do not include individuals over 65 years of age. It has been shown that climacteric symptoms influence female sexual function, significantly increasing the occurrence of sexual dysfunction and affecting the quality of life of middle-aged women [44]. In fact Cruz et al. [46], in a study of Brazilian women aged 40–65 years, showed that the intensity of climacteric symptoms was associated with the presence of sexual dysfunction. Our results are in agreement with theirs, but in addition we found that severe MRS symptoms were associated with decreased female sexual function among middle-aged and older women. Pain was the only FSFI domain that was not associated with the impact of menopausal symptoms on quality of life, which is in line with observations reported by Nazarpour et al. [35] for postmenopausal women (52.8 ± 3.7 years). They were also unable to find any association between quality of life and the pain domain of the FSFI.

Previous research has reported significant associations between sexual dysfunction and urogenital symptoms [39], and Chedraui et al. [45] suggested that sexual function was highly influenced by the urogenital function. The findings of the present study support these results, as the urogenital domain of MRS was the one displaying more associations with FSFI in the multivariate analysis. In fact this was, along with age, the only factor that retained an association with the lubrication subscale of FSFI. Low vaginal blood supply, associated with decreased estrogen levels, affects vaginal lubrication, which has been reported to be the most common cause of sexual dysfunction among middle-aged women [39,47].

It has been shown that depression may increase the occurrence of other psychological symptoms during menopause [48], even in individuals without a history of depression. Depressive symptoms, as well as most antidepressants, have been shown to be associated with a higher risk of decreased sexual desire [49]. A study performed on healthy adult women across the United States showed that women with depression were twice as likely to experience lower levels of desire, arousal, and orgasmic function [50]. Our research suggests that depression was individually associated with lower levels of desire and arousal, as well as with poorer scores in the FSFI lubrication subscale and FSFI total score, but these associations disappeared in the multivariate analysis. Nevertheless, the results of our multivariate analysis are consistent with this previous work and show that the severity of psychological symptoms was linked to lower arousal and orgasm scores, while a severe MRS total score (at the expense of the MRS urogenital and psychological scores) was associated with lower desire levels.

Some limitations of this study should be acknowledged. To begin with, it was performed on women recruited from a specific geographical area, and any generalization of its results should be limited to individuals of similar characteristics. Secondly, the cross-sectional design of this research does not allow us to establish causal relations between menopause symptoms and female sexual function. Another limitation is the lack of assessment of partner-related variables, any medication that may affect sexual function, or a hormonal profile, since some hormones play an important role in sexual function. A measure of sexual distress was not included and it may have resulted in a higher estimation of female sexual dysfunction [51]. Lastly, only 13 patients were included in the depressed mood group and the results in this subgroup must be taken carefully because there is a big difference in number compared with the group of women without depressed mood. Future studies should explore prospective designs for a larger and more diverse population, including women with a variety of health conditions, stratifying participants by age groups, and taking into account sexual distress, hormonal profile, partner-related variables and the use of any medication with the potential to affect sexual function.

## 5. Conclusions

Our study showed that, besides confounders such as older age, urban area of residence, and not working status, the severe impact of menopausal symptoms is associated with decreased female sexual function among middle-aged and older postmenopausal women. More specifically, the multivariate analysis revealed that severe psychological menopausal symptoms were linked to the arousal, orgasm, and overall FSFI scores, while severe menopause-related urogenital symptoms were associated with all FSFI total scores except for pain and desire. The latter was also linked to the MRS total score. Therefore, menopausal symptoms should be considered when analyzing female sexual function and devising strategies for its improvement among middle-aged and older postmenopausal women.

## Figures and Tables

**Table 1:** Descriptive data of the sample.

Characteristics		Participants (*n* = 182)			
		Mean	SD	Frequency	Percentage
Age (years)		65.59	7.93		
Occupational status	Not working			115	63.19
	Working			67	36.81
Education	Primary or less			109	59.89
	Secondary or higher			72	39.56
Area of residence	Rural			46	25.27
	Urban			136	74.73
Depression (HADS)	Yes			13	7.14
	No			169	92.86
MRS	Somatic	5.31	3.01		
	Psychological	4.02	3.46		
	Urogenital	3.74	3.28		
	Total score	13.07	7.52		
FSFI	Desire	2.77	1.55		
	Arousal	2.72	1.91		
	Lubrication	2.90	1.93		
	Orgasm	3.08	2.10		
	Satisfaction	3.57	1.76		
	Pain	3.18	2.14		
	Total score	18.22	10.60		

SD: Standard deviation. FSFI: Female Sexual Function Index. HADS: Hospital Anxiety and depression Scale. MRS: Menopause Rating Scale.

**Table 2:** Female sexual function according to the MRS domains and total score groups (*n* = 182).

FSFI	MRS Somatic					MRS Psychological					MRS Urogenital					MRS Total				
	Non-Severe (*n* = 155)		Severe (*n* = 27)		*p*-Value	Non-Severe (*n* = 145)		Severe (*n* = 37)		*p*-Value	Non-Severe (*n* = 100)		Severe (*n* = 82)		*p*-Value	Non-Severe (*n* = 125)		Severe (*n* = 57)		*p*-Value
Desire	2.82	1.60	2.51	1.18	0.245	2.95	1.53	2.06	1.40	0.002	3.10	1.55	2.37	1.46	0.001	2.96	1.57	2.36	1.42	0.014
Arousal	2.69	1.99	2.89	1.45	0.540	2.92	1.90	1.95	1.80	0.006	3.14	1.88	2.21	1.84	0.001	2.88	1.95	2.36	1.79	0.090
Lubrication	2.87	2.01	3.09	1.40	0.482	3.04	1.90	2.34	1.99	0.049	3.35	1.84	2.35	1.91	<0.001	3.01	1.96	2.66	1.87	0.266
Orgasm	3.03	2.17	3.36	1.69	0.367	3.27	2.07	2.30	2.07	0.012	3.54	2.04	2.52	2.05	0.001	3.25	2.14	2.70	1.99	0.104
Satisfaction	3.53	1.81	3.85	1.45	0.306	3.70	1.75	3.06	1.75	0.047	3.86	1.71	3.22	1.78	0.015	3.67	1.77	3.35	1.75	0.259
Pain	3.08	2.18	3.79	1.81	0.074	3.21	2.06	3.08	2.47	0.774	3.39	2.00	2.93	2.28	0.150	3.18	2.09	3.19	2.27	0.964
Total score	18.00	11.04	19.50	7.63	0.389	19.10	10.50	14.80	10.40	0.027	20.38	10.22	15.60	10.52	0.002	18.95	10.78	16.63	10.11	0.172

Values expressed as means and standard deviations. FSFI: Female Sexual Function Index. MRS: Menopause Rating Scale.

**Table 3:** Female sexual function according to the depression (*n* = 182).

FSFI	Depression (HADS)				
	No (*n* = 169)		Yes (*n* = 13)		*p*-Value
Desire	2.85	1.53	1.75	1.46	0.013
Arousal	2.81	1.90	1.55	1.80	0.021
Lubrication	2.99	1.92	1.66	1.76	0.016
Orgasm	3.15	2.08	2.18	2.25	0.112
Satisfaction	3.64	1.74	2.77	1.91	0.088
Pain	3.23	2.11	2.62	2.49	0.323
Total score	18.66	10.48	12.53	10.88	0.044

Values expressed as means and standard deviations. FSFI: Female Sexual Function Index. HADS: Hospital Anxiety and Depression Scale.

**Table 4:** Multivariate linear regression analyses for variables associated with FSFI scores (*n* = 182).

FSFI	Independent Variables	B	β	t	IC 95%		Multiple R	R^2^	Adjusted R^2^	*p*-Value
Desire	Age	−0.113	−0.580	−9.773	−0.135	−0.090	0.584	0.341	0.337	<0.001
	MRS total score severity	−0.587	−0.178	−2.995	−0.974	−0.200	0.604	0.365	0.358	0.003
Arousal	Age	−0.125	−0.518	−7.681	−0.157	−0.093	0.686	0.471	0.468	<0.001
	Urban area of residence	−1.471	−0.334	−6.648	−1.907	−1.034	0.730	0.533	0.528	<0.001
	MRS urogenital severity	−0.529	−0.138	−2.741	−0.911	−0.148	0.745	0.555	0.547	0.007
	MRS psychological severity	−0.811	−0.172	−3.474	−1.271	−0.350	0.752	0.565	0.556	0.001
	Not working	−0.907	−0.230	−3.365	−1.439	−0.375	0.778	0.605	0.594	0.001
Lubrication	Age	−0.146	−0.600	−10.232	−0.174	−0.118	0.641	0.411	0.405	<0.001
	MRS urogenital severity	−0.526	−0.136	−2.325	−0.973	−0.080	0.652	0.425	0.415	0.021
Orgasm	Age	−0.133	−0.504	−6.818	−0.172	−0.095	0.623	0.388	0.385	<0.001
	Urban area of residence	−1.705	−0.353	−6.412	−2.229	−1.180	0.680	0.462	0.456	<0.001
	MRS urogenital severity	−0.699	−0.166	−3.012	−1.157	−0.241	0.617	0.381	0.377	0.003
	MRS psychological severity	−0.807	−0.156	−2.877	−1.360	−0.253	0.670	0.449	0.443	0.005
	Not working	−0.683	−0.158	−2.108	−1.322	−0.044	0.725	0.526	0.512	0.036
Satisfaction	Age	−0.139	−0.629	−11.165	−0.164	−0.115	0.508	0.258	0.254	<0.001
	Urban area of residence	−1.187	−0.293	−5.128	−1.643	−0.730	0.588	0.346	0.339	<0.001
	MRS urogenital severity	−0.434	−0.123	−2.130	−0.835	−0.032	0.607	0.368	0.357	0.035
Pain	Age	−0.108	−0.403	−4.850	−0.152	−0.064	0.653	0.426	0.423	<0.001
	Urban area of residence	−1.598	−0.325	−5.323	−2.190	−1.005	0.708	0.501	0.495	<0.001
	Not working	−0.917	−0.208	−2.475	−1.648	−0.186	0.607	0.368	0.357	0.014
Total score	Age	−0.676	−0.508	−7.128	−0.863	−0.489	0.584	0.341	0.337	<0.001
	Urban area of residence	−8.563	−0.352	−6.631	−11.112	−6.015	0.604	0.365	0.358	<0.001
	MRS urogenital severity	−2.950	−0.139	−2.616	−5.175	−0.724	0.686	0.471	0.468	0.010
	Not working	−4.477	−0.205	−2.845	−7.582	−1.371	0.736	0.542	0.531	0.005
	MRS psychological severity	−3.578	−0.137	−2.627	−6.267	−0.890	0.745	0.555	0.547	0.009

B: Unstandardized Coefficient. β: Standardized Coefficient. CI: Confidence Interval. FSFI: Female Sexual Function Index. MRS: Menopause Rating Scale. R^2^: Coefficient of determination.
